# Mechanical Characterization of 3D-Printed Scaffolds: A Multi-Objective Optimization Approach Using Virtual Testing and Homogenization

**DOI:** 10.3390/biomimetics10090580

**Published:** 2025-09-02

**Authors:** Pablo I. León, Uwe Muhlich, Pedro C. Aravena, Gabriela Martínez

**Affiliations:** 1 Programa de Magister en Ingeniería Mecánica y Materiales, Facultad de Ciencias de la Ingeniería, Universidad Austral de Chile, Valdivia 5090000, Chile; pablo.leon@alumnos.uach.cl; 2 Instituto de Obras Civiles, Facultad de Ciencias de la Ingeniería, Universidad Austral de Chile, Valdivia 5090000, Chile; uwe.muhlich@uach.cl; 3 Escuela de Odontología, Facultad de Medicina, Universidad Austral de Chile, Valdivia 5090000, Chile; paravena@uach.cl; 4 Instituto de Ingeniería Mecánica, Facultad de Ciencias de la Ingeniería, Universidad Austral de Chile, Valdivia 5090000, Chile

**Keywords:** 3D-printed scaffolds, homogenization, virtual testing, multi-objective optimization, mechanical properties, tissue engineering

## Abstract

A method to characterize the mechanical properties of cellular materials manufactured using 3D printing, specifically employing the fused deposition modeling (FDM) technique, is developed. Numerical simulations, virtual testing, and optimization based on genetic algorithms are combined in this approach to determine the anisotropic properties of the material, which are essential for biomedical applications such as tissue engineering. Homogenization using representative unit cells enabled the calculation of orthotropic properties, including elastic moduli ($E_{1}$, $E_{2}$, $E_{3}$), Poisson’s ratios ($\nu_{12}$, $\nu_{13}$ and $\nu_{23}$), and shear moduli ($G_{12}$, $G_{13}$, $G_{23}$). These results validated the virtual tests using an L-shaped beam model, based on a known state of displacements and stresses. In the virtual test of the FDM model, a significant correlation with experimental results was observed, confirming the material’s anisotropy and its influence on deformations and stresses. Meanwhile, the effective medium test demonstrated over 95% agreement between simulated and experimental values, validating the accuracy of the proposed constitutive model. The optimization process, based on multi-objective genetic algorithms, allowed the determination of the material’s mechanical properties through controlled iterations, achieving a strong correlation with the results obtained from the homogenization model. These findings present a new approach for characterizing and optimizing 3D-printed materials using FDM techniques, providing an efficient and reliable method for applications in tissue engineering.

## 1. Introduction

Scaffolds are solid and porous matrices that, when loaded with cells, are used to guide the development of new tissue by providing mechanical stability and allowing cell adhesion and proliferation. Their structural properties, including external geometry, porosity, interconnectivity, pore size, and surface area, are considered key parameters in determining their effectiveness [1]. Geometry plays a fundamental role in the bone growth process. Ensuring scaffold interconnectivity is essential, as it facilitates cell migration and tissue development throughout the entire structure. In this regard, various geometric patterns have been proposed and evaluated, including rectangular and triangular patterns [2,3], and more recently, triply periodic minimal surface (TPMS) structures [4,5], with particular attention given to gyroid patterns [6].

From a biomechanical perspective, scaffold stiffness is considered a crucial mechanical property, as it is used to quantify how closely the scaffold matches the stiffness of the surrounding bone. For instance, the elastic modulus of trabecular bone has been reported to range between 0.1 and 2 GPa, while that of cortical bone varies between 15 and 20 GPa. However, although porosity is essential for cell growth and vascularization, it has been shown to reduce the mechanical properties of the scaffold [7].

Although stiffness is considered an important factor in structural response, pore size and porosity are also regarded as key determinants [8,9]. While an increase in porosity has been shown to reduce mechanical properties, it is essential for facilitating cell growth and scaffold vascularization [10]. Significant controversy has arisen regarding the appropriate porosity levels and pore sizes for these structures, primarily due to the heterogeneity of bone tissue depending on the anatomical location. Conservative ranges have been suggested, indicating that porosity above 50% is recommended for scaffold design [11]; however, porosities as low as 30% have also been studied. Particularly, Aydin et al. (2024) [12] suggest a porosity of 70% and a pore size of 600 nm, which closely approximates the natural porosity of bone [12]. These dimensions facilitate macrophage infiltration and promote the influx of growth factors essential for tissue colonization, cell migration, and in vivo vascularization [13]. In compact bone, Haversian and Volkmann canals (20–100 μm) are essential for metabolic activity [14,15]. In contrast, trabecular bone contains larger pores, which facilitate nutrient exchange. Significant variability has been reported, with studies indicating pore sizes ranging from 100 to 1250 nm [2,16,17].

Diverse fabrication techniques have been developed to manufacture complex porous structures for bone tissue engineering, including thermally induced phase separation (TIPS), electrospinning, and additive manufacturing (AM). TIPS enables the creation of highly porous and interconnected polymer matrices through solvent crystallization and phase separation, resulting in scaffolds with favorable characteristics for cell attachment, proliferation, and nutrient diffusion [18,19]. Electrospinning allows the fabrication of nanofibrous mats that closely mimic the architecture of the extracellular matrix, thereby enhancing osteogenic potential [20,21]. However, both techniques present limitations in terms of structural reproducibility and scalability, particularly when aiming to fabricate patient-specific constructs or mechanically optimized architectures.

Additive manufacturing (AM), particularly fused deposition modeling (FDM), stands out for its accessibility, cost-efficiency, and ability to process biocompatible materials such as polylactic acid (PLA), a biodegradable polymer widely used in tissue engineering [22]. Moreover, AM techniques can be directly integrated with computational design and simulation workflows, facilitating optimization and virtual testing. Despite these advantages, scaffolds fabricated via FDM exhibit mechanical anisotropy due to the layer-by-layer deposition process and printing parameters such as filament orientation, infill density, and layer spacing [23]. These factors significantly influence scaffold performance and must be considered to ensure functionality under physiological conditions.

A major challenge is the lack of standardized methods for mechanically characterizing polymer components manufactured using FDM [24,25]. Previous research has adapted standards such as ASTM D638 [26] and ASTM D3039 [27] to evaluate the mechanical properties of 3D-printed materials; however, these standards do not account for the anisotropic microstructure of the printed parts, leading to inconsistencies in the results [24,25]. Furthermore, failures observed in specimens during tensile testing, such as stress concentrations in the gripping areas, have highlighted the need to develop specific standards for FDM samples [23].

Considering the cost and complexity of experimental testing, virtual testing via finite element analysis (FEA) provides an efficient method to predict scaffold behavior and optimize design. Finite element analysis (FEA) is widely used to assess scaffold performance. Soufivand et al. (2020) [28] estimated compressive elastic moduli for various printing patterns, while Kladovasilakis et al. (2023) [29] validated FEM simulations of tibial scaffolds against experimental data, identifying stress concentrations and supporting in vivo viability. Additionally, FEM updating techniques enable inverse identification of material parameters by comparing simulations with experimental displacements.

Optimization techniques play a crucial role in the mechanical characterization and design of 3D-printed components. The evolution of these methods has advanced significantly since the 1980s, when Kirkpatrick et al. (1983) [30] introduced *Simulated Annealing*, a seminal contribution that marked a turning point in the development of heuristics inspired by physical processes. Subsequently, genetic algorithms introduced by Goldberg (1989) [31], along with multi-objective frameworks such as the *Non-dominated Sorting Genetic Algorithm II* (NSGA-II) [32] and the *Multi-Objective Genetic Algorithm* (MOGA) [33,34], expanded the applicability of these approaches to complex problems involving multiple constraints and non-trivial solution spaces. Broadly speaking, optimization methods can be classified according to their structural nature (deterministic vs. stochastic) and their guiding paradigm (conventional, heuristic, or metaheuristic) [35]. Among the metaheuristic class, evolutionary algorithms have demonstrated effectiveness in addressing highly nonlinear, multi-objective problems—such as the estimation of orthotropic material parameters in systems with intricate microstructures. Thus, they represent a valuable tool with demonstrated applicability in inverse mechanical property identification.

Another technique used to predict the effective macroscopic properties of 3D-printed structures is homogenization, which relies on the properties of the constituent material and the characteristic geometry of the microstructure. Gonabadi et al. (2022) [36] conducted experimental, microstructural, and homogenization-based tests on 3D-printed parts with different infill densities and concluded that homogenization is a reliable tool for predicting the elastic response. This project aims to determine the mechanical properties of a material fabricated using the fused deposition modeling (FDM) technique, considering the printing parameters defined based on scaffold design. To achieve this, the development of a virtual mechanical test is proposed, integrating multiple relevant deformation states to enable the indirect characterization of the material’s properties through finite element analysis (FEA) combined with optimization techniques. The obtained results will be validated by comparison with homogenization models of representative unit cells. Although different commercial tools were used during the development of this study, the originality of the proposed methodology lies in the integration of a complete virtual workflow, combining unit cell modeling, mechanical homogenization, optimization strategies, and the definition of an admissible design space. This methodology enables efficient virtual characterization and optimization of scaffold structures, minimizing the need for preliminary experimental testing.

## 2. Methodology

**Figure 1:**
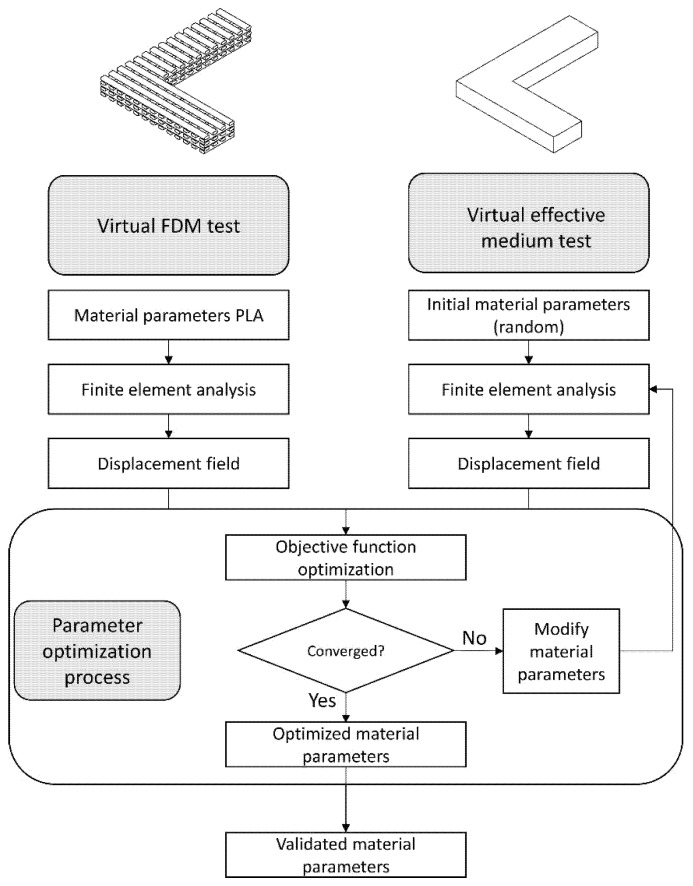
General schematic of the evaluation procedure for the proposed methodology.

A virtual test was developed using the finite element method (FEM), employing an L-shaped beam that integrates critical geometric parameters from the design region, including pore size, layer height, and porosity. From this model, characteristic displacement fields are obtained. Subsequently, a second virtual test is performed using a fully solid L-shaped beam. Through the application of multi-objective optimization techniques, the properties of the solid model are iteratively adjusted until convergence with the displacement fields of the initial model is achieved, enabling the determination of the effective mechanical properties. Finally, the obtained results are validated through homogenization techniques, allowing for a comparative analysis and verification of the effective properties in relation to the microstructural characteristics of the fabricated components. The entire process is illustrated in Figure 1.

### 2.1. Design Zone

The characterization process begins with the definition of the scaffold’s design zone, considering parameters such as porosity, pore size, interconnectivity, and infill pattern. These parameters are essential to ensure appropriate mechanical behavior and optimal biological functionality. The efficiency of the scaffold is directly linked to the structural properties of the representative unit cell, including topology, interconnectivity, and constituent material [11].

In this study, FDM was selected as the manufacturing technique, and PLA was chosen due to its low cost and biocompatibility. It has been concluded by Alonso-Fernández et al. (2023) [37] that FDM combined with PLA represents a promising option for the fabrication of scaffolds intended for the treatment of bone defects [37].

**Figure 2:**
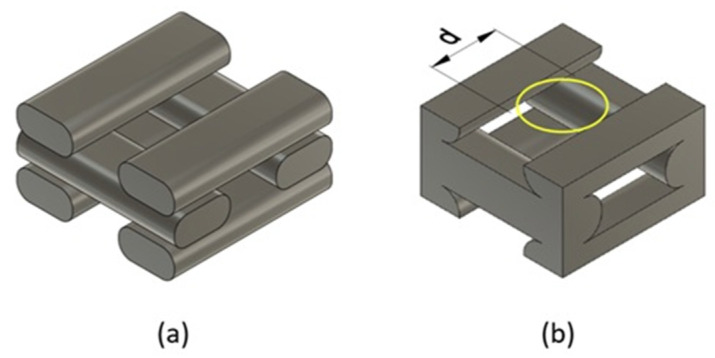
(**a**) Rectilinear printing pattern (0°/90°); (**b**) Unit cell associated with the printing pattern.

One of the most critical parameters in the FDM technique is the infill pattern. In this work, a rectilinear filament pattern (0°/90°) was selected, as it promotes pore interconnectivity. Although alternative geometries such as gyroid or triangular patterns have demonstrated greater mechanical efficiency and improved pore interconnectivity [4,5,6], a rectilinear (0°/90°) pattern was employed in this study due to its simplicity, ease of parametric control, and frequent use in FDM printing applications. This pattern enables a clear definition of orthotropic axes, which is essential for the validation of the proposed constitutive model. Additionally, the rectilinear configuration ensures consistent filament deposition and a predictable structure, allowing for reproducible simulation and homogenization analyses. Figure 2a illustrates this pattern, while Figure 2b shows the associated unit cell.

**Figure 3:**
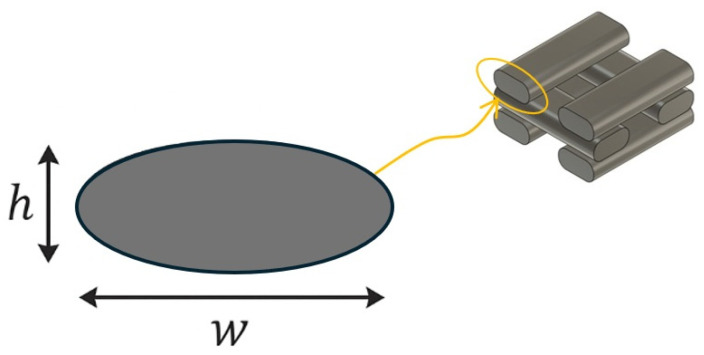
Representation of the ellipsoidal geometry of the filament deposited by FDM.

The fused deposition modeling (FDM) process results in an ellipsoidal cross-sectional geometry of the extruded filaments, as previously reported by other authors [36]. Figure 3 provides a schematic illustration of the filament cross-section, depicting a simplified ellipsoidal profile. The dimensions *w* and *h* represent the filament width and layer height, respectively. This geometric approximation is considered in the porosity calculation to account for the actual deposition morphology.

Porosity is measured based on the percentage of void space in a fully solid cell, according to the following equation [38]:

(1) $$\mathrm{Porosity}\left(\%\right)=\left(1-\frac{V_{p}}{V_{s}}\right)\;x\;100$$

where:

$V_{p}$: Volume of the porous unit cell.

$V_{s}$*:* Volume of the solid unit cell.

The pore size was determined using the largest inscribed circle method [11], as shown in Figure 2b. This parameter is related to the infill density, as a larger pore size corresponds to a greater distance between printed filaments.

**Figure 4:**
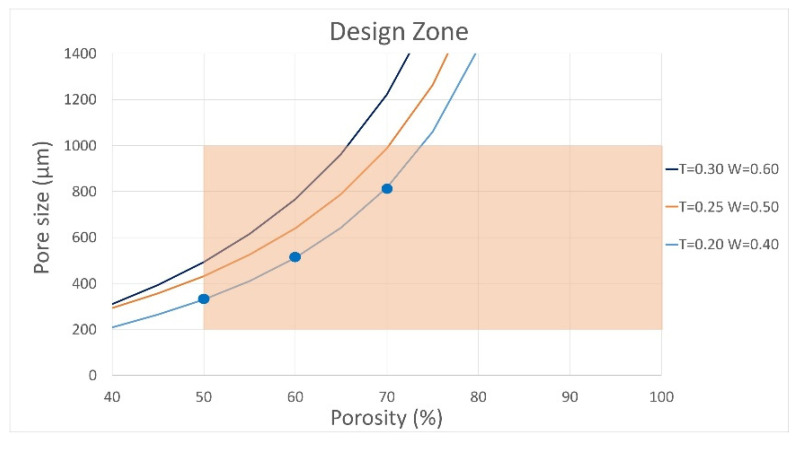
Admissible design zone with porosity and pore size constraints. No experimental testing was performed.

Following the selection of the printing geometry, an admissible design space was defined to evaluate representative combinations of pore size and porosity, ensuring both biological relevance and manufacturability. Figure 4 shows this design space, with porosity on the *x*-axis and pore size on the *y*-axis. The three curves correspond to filaments printed with nozzle diameters (W) of 0.4, 0.5, and 0.6 mm, and layer heights (T) of 0.20, 0.25, and 0.30 mm, calculated based on filament spacing under different printing conditions. Biological constraints were established from previous studies recommending a porosity greater than 50% [11,39] and pore sizes between 200 µm and 1 mm, optimal for bone regeneration [40,41]. Within these limits, the admissible zone (depicted in orange) ensures compliance with both biological requirements and FDM manufacturing constraints.

The selected study points correspond to three pore sizes and porosities within the admissible design zone. These test cases are presented in Table 1. The three test cases were selected based on standard FDM printing practices using a 0.4 mm nozzle. These test cases cover a practical range of layer heights and filament widths, enabling the analysis of scaffold structures with porosities above 50% and pore sizes within biologically relevant ranges for tissue engineering applications.

It should be noted that the pore size was predefined within the biologically admissible range (200 µm to 1 mm) and was not treated as a variable in the optimization process. The optimization was exclusively focused on adjusting the effective mechanical properties associated with each predefined geometric test case, without modifying the initial pore size or porosity values.

### 2.2. Constitutive Model

The mechanical behavior of FDM-printed parts is modeled assuming that unit cell properties remain constant with varying layer heights, provided porosity is preserved—a premise supported by prior studies [36], which identify porosity as the primary factor influencing effective stiffness. Test cases such as 0.2/0.4, 0.25/0.5, and 0.3/0.6 mm, maintaining equal solid volume fractions, are thus expected to exhibit similar orthotropic responses under consistent filament architectures. The 0.2/0.4 mm case was selected as the reference due to its prevalence in FDM and its representativeness of biologically relevant pore sizes. Despite the potential for local mechanical variations at interlayer and inter-filament regions, a uniform material behavior at the unit cell level was assumed to enable homogenization, in line with established methodologies [11,36]. Structural behavior was simulated using FEM: isotropic properties were assigned to bulk PLA, while orthotropic linear elasticity was assumed for cellular scaffolds. Although nonlinear and anisotropic effects have been reported [42], this study focuses on linear elastic response as a valid approximation for micromechanical modeling. An orthotropic material is characterized by three symmetrical planes that coincide with the coordinate planes, and its behavior is described by nine elastic constants. The relationship between stress and small deformations is formulated based on Hooke’s law, as presented in Equation (2) [43].

(2) $$\sigma_{ij}=C_{ij}\cdot \varepsilon_{ij}$$

where $C_{ij}$ represents the elements of the stiffness matrix, $\varepsilon_{ij}$ denotes the strain components, and $\sigma_{ij}\;$corresponds to the associated stresses. By inverting the equation, the flexibility matrix $S_{ij}$ is obtained, which allows the calculation of the engineering constants through the following relationship:

(3) $$\varepsilon_{ij}=S_{ij}\cdot \sigma_{ij}$$

The engineering constants, including the Young’s moduli ($E_{1}$, $E_{2}$, $E_{3}$), the shear moduli ($G_{12}$, $G_{13}$, $G_{23}$), and the Poisson’s ratios ($\nu_{12}$, $\nu_{13}$, $\nu_{23}$), can be calculated by developing the flexibility matrix:(4)
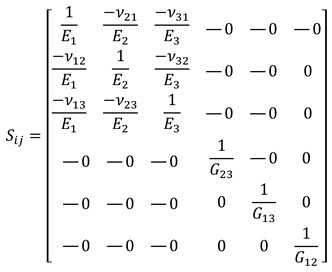


Previous studies have indicated equality in some elastic parameters depending on the printing direction [39,44], specifically:

(5) $$E_{1}=E_{3}$$

(6) $$G_{12}=G_{23}$$

In this way, the orthotropic material is described using seven constants: $E_{1}$, $E_{2},G_{12}$, $G_{13},\nu_{12}$, $\nu_{13}$ and $\nu_{23}$. This approach will be used to define the orthotropic behavior of the geometries under study.

### 2.3. Homogenization Methodology

Homogenization refers to the process of replacing a heterogeneous material, such as those composed of complex microstructures, with an equivalent homogeneous medium that approximates its macroscopic mechanical behavior. The general concept has been developed within the framework of multiscale analysis, where the behavior of materials with periodic or quasi-periodic microstructures is described using asymptotic expansions and averaging methods [45]. These methods aim to relate small-scale structural features to effective large-scale properties.

In periodic media, it is commonly assumed that the global response of a composite material can be captured by a representative volume element, also referred to as a unit cell. This cell contains the essential geometrical and material information needed to estimate the effective properties. While this study adopts a numerical and engineering-focused approach, it aligns conceptually with the theoretical framework described above. In particular, the methodology relies on volume averaging, periodic boundary conditions, and mechanical simulations of a unit cell to estimate effective elastic moduli.

**Figure 5:**
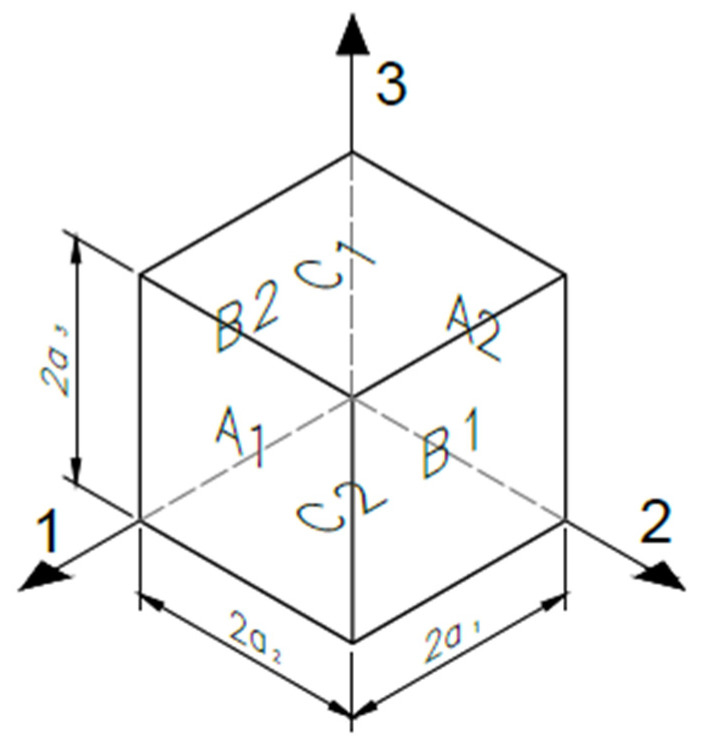
Unit cell model showing orientation surfaces and boundaries.

In this work, the homogenization procedure was fully implemented using the Material Designer module of ANSYS^®^ (2023) [33], which automates the steps of geometry definition, mesh generation, material assignment, application of periodic boundary conditions, and mechanical analysis. Unlike asymptotic-based homogenization theories, this tool adopts a purely computational approach, relying on periodic boundary conditions and stress-strain averaging over a representative volume element, without requiring explicit analytical developments. The scaffold was modeled as a periodic medium in the three spatial directions, with the unit cell defined by parameters such as porosity, pore size, and layer height [39]. The PLA material was considered isotropic at the filament scale, while anisotropy at the scaffold level results from the internal architecture and printing pattern. The module outputs the homogenized orthotropic elastic constants derived from the unit cell’s response under six independent load cases. The unit cell configuration and its boundary surfaces are illustrated in Figure 5.

A representative unit cell, defined by parameters such as porosity, pore size, and layer height, was modeled assuming periodic repetition in all spatial directions [39]. To compute the global stiffness matrix C, average strains ($\varepsilon_{ij}^{0}$) were applied using periodic boundary conditions proposed by Luciano and Sacco (1998) [46], ensuring translational symmetry:

(7) $$u_{i}\left(a_{1},x_{2},x_{3}\right)-u_{i}\left(a_{1},x_{2},x_{3}\right)=2a_{1}\varepsilon_{i1}^{0}$$

(8) $$u_{i}\left(x_{1},a_{2},x_{3}\right)-u_{i}\left(x_{1},a_{2},x_{3}\right)=2a_{2}\varepsilon_{i2}^{0}$$

(9) $$u_{i}\left(x_{1},x_{2},a_{3}\right)-u_{i}\left(x_{1},x_{2},a_{3}\right)=2a_{3}\varepsilon_{i3}^{0}$$

The relationship between the average stress ${\bar{\sigma }}_{ij}$ and the imposed average strain $\varepsilon_{\mathrm{ij}}^{0}$ is expressed as:

(10) $${\bar{\sigma }}_{ij}=C_{ij}\varepsilon_{\mathrm{ij}}^{0}$$

where $C_{ij}$ represents the effective stiffness tensor of the equivalent homogeneous material.

The average stress ${\bar{\sigma }}_{ij}$ is computed as the volume average of the local stress field over the total volume V of the representative volume element [47]:

(11) $${\bar{\sigma }}_{ij}=\frac{1}{V}\int_{v}\;\sigma_{ij}dv$$

Similarly, the average local strain ${\bar{\varepsilon }}_{ij}\;$is calculated as the spatial average of the local strain field, and its relation to $\varepsilon_{\mathrm{ij}}^{0}$ depends on the boundary conditions imposed during the homogenization analysis.

Each component $C_{ij}$ of the stiffness matrix was computed by applying a specific deformation mode (e.g., $\varepsilon_{11}^{0}\;=\;0.01$, others = 0) and using the relation:

(12) $$C_{\alpha 1}=\frac{P_{\alpha }}{S_{\alpha }*0.01}$$

where $P_{\alpha }$ is the reaction force and $S_{\alpha }$ is the loaded surface area. This procedure was repeated for all components of $C_{\alpha i}$, applying controlled strain states [48]. The displacement conditions for each case are summarized in Table 2.

### 2.4. Optimization Methodology

While optimization techniques have been widely applied to the geometric design of scaffolds for tissue engineering [49,50,51], their use for estimating effective mechanical properties remains limited. In this section, a strategy based on MOGA is proposed to fit the orthotropic elastic constants of the constitutive model, validating its effectiveness through comparison with reference FDM models. To achieve this an experimental test is designed to determine the elastic constants of a part fabricated using FDM with properties required for its use as a scaffold. This is achieved through an optimization process in which the material’s elastic constants are varied to meet certain imposed constraints. The constraints correspond to variables that can be measured in an experimental test, such as displacements, rotations, or determined forces. The selection of these constraints is made through a sensitivity study, in which their relevance for determining the material’s elastic constants is evaluated. Although the present study adopts a Multi-Objective Genetic Algorithm (MOGA) framework, it is important to acknowledge the broader context of metaheuristic optimization methods. The foundational work of Kirkpatrick et al. (1983) [30] on simulated annealing introduced key concepts that influenced the development of subsequent heuristic strategies, including evolutionary algorithms like MOGA [30,52]. These methodologies have enabled the exploration of complex design spaces, such as the one addressed in this study.

To achieve this, two virtual tests are performed, namely:

1. Virtual test of the FDM model: This test simulates a part fabricated using 3D printing.
2. Virtual test of the effective medium: This test considers the previously fabricated part as an effective medium model.

Both models are shown in Figure 1. In this process, certain variables are measured in the FDM model, which are then used as constraints to perform the optimization in the effective medium model, which adjusts its elastic constants to meet the imposed constraints.

In this evaluation, the ANSYS^®^ Design Xplorer 2023 tool is used; the selected direct optimization method is the multi-objective genetic algorithm (MOGA) [33]. The workflow steps of the selected optimization method are as follows:

1. Initialization: The first population is generated randomly within the defined bounds.
2. Evaluation: Each design point is simulated, and objective functions are computed.
3. Reproduction: New populations are generated via crossover and mutation.
4. Convergence validation: Checks whether Pareto stability or convergence thresholds are met.
5. Stopping criteria validation: If convergence is not achieved, the algorithm continues unless the maximum number of iterations is reached.
6. Finalization: The optimization ends when convergence or stopping criteria are satisfied.

Once convergence is reached, MOGA delivers three candidate solutions. Candidate 1 is prioritized, subject to two key validations:

Sensitivity Validation: The influence of each constraint (input) on the estimated parameters (outputs) is analyzed using the sensitivity analysis tools in ANSYS DesignXplorer. This includes sensitivity coefficients and tornado plots, which indicate the degree to which each input variable affects the outputs. If selected constraints exhibit low sensitivity, they may be discarded or replaced by alternative, more informative measures.

Range Validation: The estimated elastic constants are checked to ensure they do not lie at the extremes of the predefined search domain. This is done by comparing the optimized values with the lower and upper bounds set initially. Solutions near the boundaries are considered less reliable, as they may indicate insufficient information in the constraints or instability in the optimization process. Only candidates whose parameters lie within a central range (e.g., 10% away from the domain limits) are accepted.

**Figure 6:**
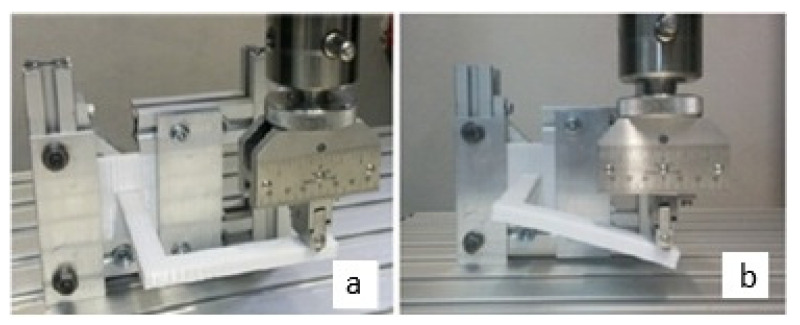
Experimental test of L-beam [53]: (**a**) initial configuration; (**b**) deformed configuration.

To validate this proposal, a design was selected considering manufacturing limitations, the feasibility of conducting the experimental test, and the existence of several relevant deformation states to obtain the elastic constants when the experimental test is performed. In this way, an L-shaped beam was selected, which was used by Domingo-Espín and his collaborators [53] to perform a result validation test, as shown in Figure 6. It is important to clarify that the L-shaped beam used in this study is not intended to replicate the geometry of a real bone scaffold, but rather serves as a simplified mechanical benchmark. Its geometry induces coupled bending and torsional responses, which are sensitive to variations in elastic constants. This allows for an evaluation of the methodology under controlled conditions.

**Figure 7:**
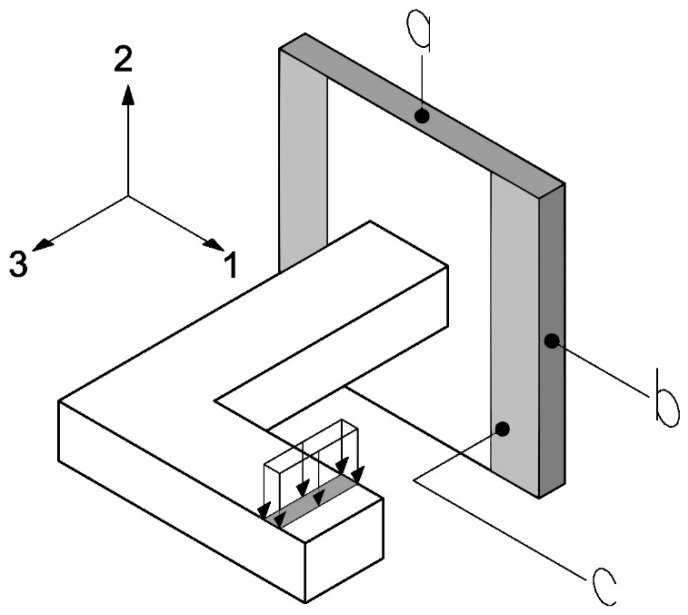
Boundary conditions in the experimental test. The surfaces a, b, and c, corresponding to the faces normal to the principal material directions 2, 1 and 3, respectively, used for the application of boundary conditions during the homogenization process.

Figure 7 shows a simplification of the previously presented L-beam test, with the loading area and boundary conditions highlighted.

The surface *a* and its opposite are subjected to the no displacement condition in the direction *2*, the surface *b* and its opposite are subjected to the no displacement condition in direction *1*, and the surface *c* is subjected to the no displacement condition in the direction *3*. Additionally, the area (near the cantilever) where a displacement of 3 mm in the negative direction of axis *2* is applied is highlighted. The conditions *a*, *b*, and *c* will be maintained throughout all tests, while the displacement conditions in the area near the cantilever may change depending on the elastic constant to be determined.

The input variables (measurements obtained from the FDM virtual test) are configured to achieve a specific target value for each parameter, which is considered to correspond to the result of its equivalent in the FDM virtual test. Additionally, upper and lower bounds are set for each input variable under the condition:

Lower Bound ≤ Value ≤ Upper Bound

These bounds are set close to the target value to reduce the number of possible combinations, thus optimizing the calculation time. A range of 10% above and below the target value is defined.

For the output variables (the elastic constants to be estimated), initial default ranges are defined. The lower bound is set to the material constant minus 10%, and the upper bound is set to the material constant plus 10%. It is noted that an orthotropic material with constantly close to the desired values is used for each test case under study.

Once convergence is achieved, MOGA delivers three candidate solutions. Candidate 1 is generally prioritized, provided it satisfies both sensitivity and range validation criteria. These validations confirm that: (a) the selected constraints meaningfully influence the optimization outcome; and (b) the estimated elastic constants lie within the central region of the predefined bounds, avoiding edge solutions that may lack physical consistency.

### 2.5. Virtual Test of Effective Medium Methodology

The virtual test of the effective medium was designed to optimize the analysis of an L-shaped beam through an iterative approach that reduces computational costs and simulation times. The main features of the test include:

- Beam dimensions: Defined according to printing limitations to ensure measurable values.
- Measurement points: Determined iteratively through sensitivity studies and measurement feasibility.

To determine the seven elastic constants, two separate tests were conducted on the same L-shaped beam. These tests were named Test 1 and Test 2 and are described below.

#### 2.5.1. Test 1

**Figure 8:**
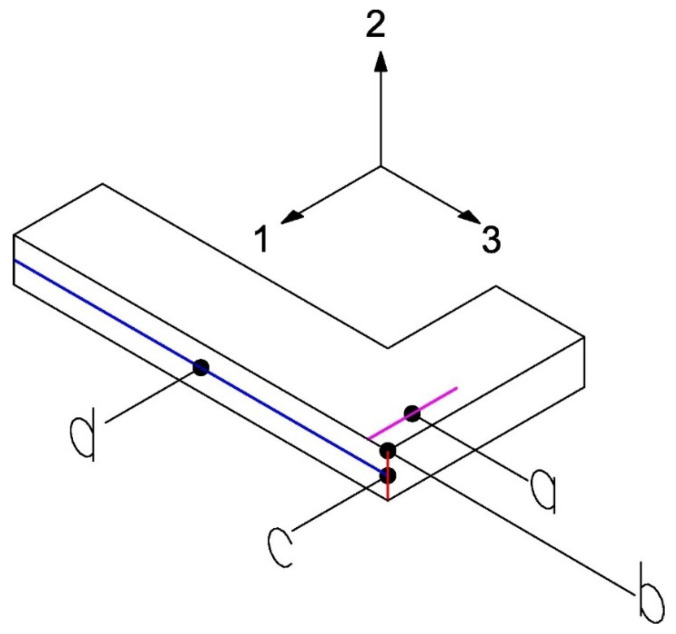
Measurements on the L-beam for Test 1.

A test configuration is used whose analytical solution is well-known and widely developed by Lechnickij in 1981 [54]. In this test, the beam is considered as an orthotropic rectangular bar subjected to bending by a transverse force (Figure 8). The elastic constants involved are $E_{3}$, $G_{13}$, $G_{23}$, $\nu_{31}$, which are obtained through the following measurements (Figure 8): the reaction force generated by applying a vertical displacement (point *a*, line pink), the displacement in the direction 2 (point *b*), the angular variation in the 2–3 plane represented by the red line (point *c*), and the average displacement along the blue horizontal line (point *d*). These measurements are integrated as constraints in the optimization process, which is performed using the MOGA method, configured to conduct approximately 3250 evaluations.

#### 2.5.2. Test 2

**Figure 9:**
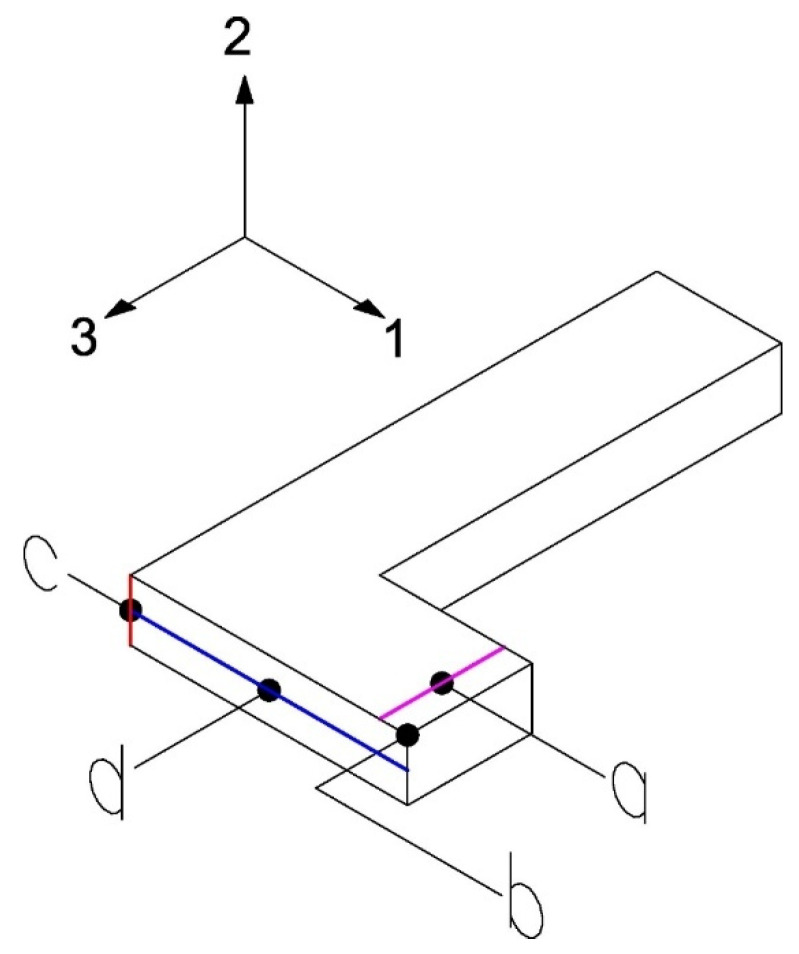
Measurements on the L-beam for Test 2.

This test applies a vertical displacement in another area of the beam (line pink, Figure 9) to generate a combined stress state and determine the constants $E_{2}$, $\nu_{12}$, $\nu_{23}$. The necessary measurements for this test are the reaction force when applying the vertical displacement (point *a*), displacement in the direction 2 (point *b*), angular variation (red line) in the 2–3 plane (point *c*), and the average displacement of the points along the blue horizontal line (point *d*). The optimization procedure is similar to that described in Test 1.

### 2.6. Virtual Test of FDM Model

**Figure 10:**
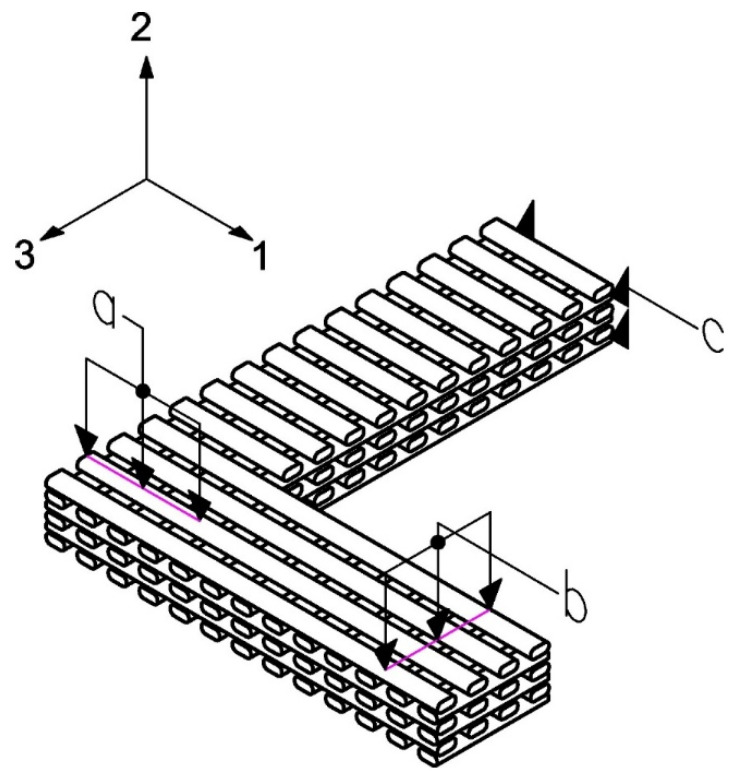
L-shaped beam that emulates the microstructure of FDM printing where *a* represents the applied force in test 1; *b* represents the applied force in test 2; *c* corresponds to the displacement restriction condition in both cases.

A microstructural simulation is conducted using the finite element method (FEM) to emulate the fabrication process via FDM printing, utilizing the ANSYS finite element software. The isotropic mechanical properties of PLA (E = 3500 MPa and ν = 0.35 [55,56,57] were assigned to the constitutive material. An L-shaped beam was created using the ANSYS Design Modeling Tool (see Figure 10). Three geometries were created corresponding to the selected design test cases shown in Table 1.

## 3. Results and Discussion

### 3.1. Homogenization Results

**Figure 11:**
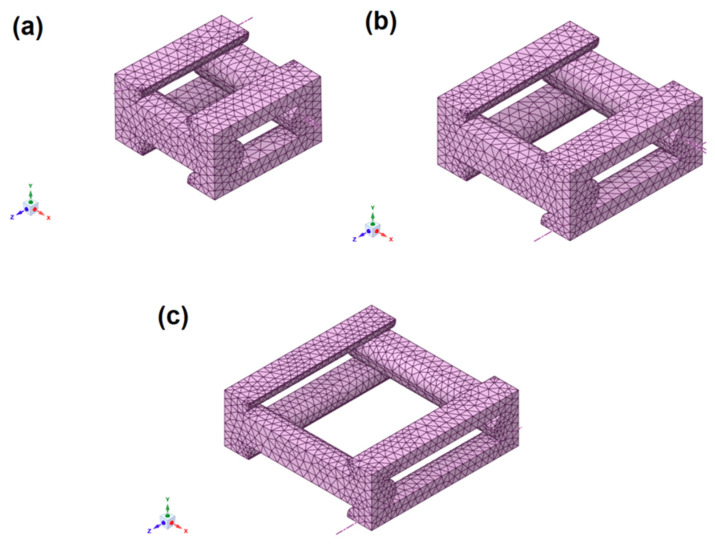
FE models of the unit cell for a 3D printed part with 0.2 mm layer height: (**a**) 50% porosity, (**b**) 60% porosity, and (**c**) 70% porosity.

The target elastic constants for the three selected test cases (see Table 1) were determined using the homogenization technique previously described. The unit cell models, generated with Ansys^®^ Material Designer [58] (Figure 11), were simulated and analyzed using an isotropic base material (*E* = 3500 MPa and *ν* = 0.35) [55,56,57], resulting in an orthotropic constitutive model and periodic boundary conditions as shown in Section 2.3.

A mesh convergence study was conducted to determine an appropriate element size for the finite element analysis. The element size of 0.07 mm was initially validated using the 50% porosity model, resulting in a relative error of less than 1% in the evaluated orthotropic properties. This element size was subsequently applied to the 60% and 70% porosity test cases, where the relative error also remained below 1%. Based on these results, an element size of 0.07 mm was adopted for all simulations

Subsequently, the elastic constants for each test case were calculated. The values obtained are presented in Table 3. These results confirmed negligible differences between $E_{1}$ and $E_{3}$, as well as between $G_{12}$ and $G_{23}$, validating the assumptions made in Equations (5) and (6). This is consistent with parts manufactured using FDM with straight filament patterns (0°/90°) and the considered porosity levels.

The results obtained through the homogenization technique reflect consistent orthotropic behavior across the three evaluated porosity configurations. Minimal differences were observed between the values of $E_{1}$ and $E_{3}$ as well as between $G_{21}$ and $G_{23}$, which supports the symmetry assumptions formulated by Bonada et al. (2021) [44] for rectilinear (0°/90°) printing patterns. This structural regularity is characteristic of parts manufactured via FDM, where the filament orientation generates preferential stiffness directions, directly affecting the material’s anisotropy [23].

Moreover, the results show a decreasing trend in the elastic and shear moduli as porosity increases, which aligns with findings by Arabnejad et al. (2016) and Zhen et al. (2010) [7,11] who demonstrated that higher porosity, while beneficial for tissue regeneration, significantly compromises the mechanical integrity of the scaffold. This behavior is consistent with the Gibson–Ashby model [59], which predicts that the elastic modulus and yield strength of cellular materials decrease as a power-law function of their relative density, following the equations:

(13) $$\frac{E^{*}}{E_{s}}=C_{1}{\left(\frac{\rho^{*}}{\rho_{s}}\right)}^{n}$$

where $\frac{\rho^{*}}{\rho_{s}}=1-P$ is inversely related to porosity P, $E_{s}$ is the elastic modulus of the solid base material, $E^{*}$ is the effective elastic modulus of the porous scaffold, $C_{1}$ is a geometric constant dependent on the unit cell topology (typically between 0.1 and 4), an n is an exponent commonly around 2 for open-cell structures. Although this relationship was originally formulated under the assumption of isotropic behavior, its decreasing trend with porosity remains valid and meaningful in the context of orthotropic scaffolds obtained through FDM, particularly when the architecture exhibits regularity and directional stiffness. Accordingly, our results confirm that increasing porosity reduces stiffness, as shown in Table 3, in agreement with this theoretical framework. In particular, the more pronounced reduction in *E*_2_ suggests that the direction orthogonal to the filaments exhibits lower structural continuity—a phenomenon also observed by Gonabadi et al. (2022) [38] in their homogenization study of FDM structures.

### 3.2. Virtual Test

#### 3.2.1. Virtual Test of FDM Model

**Figure 12:**
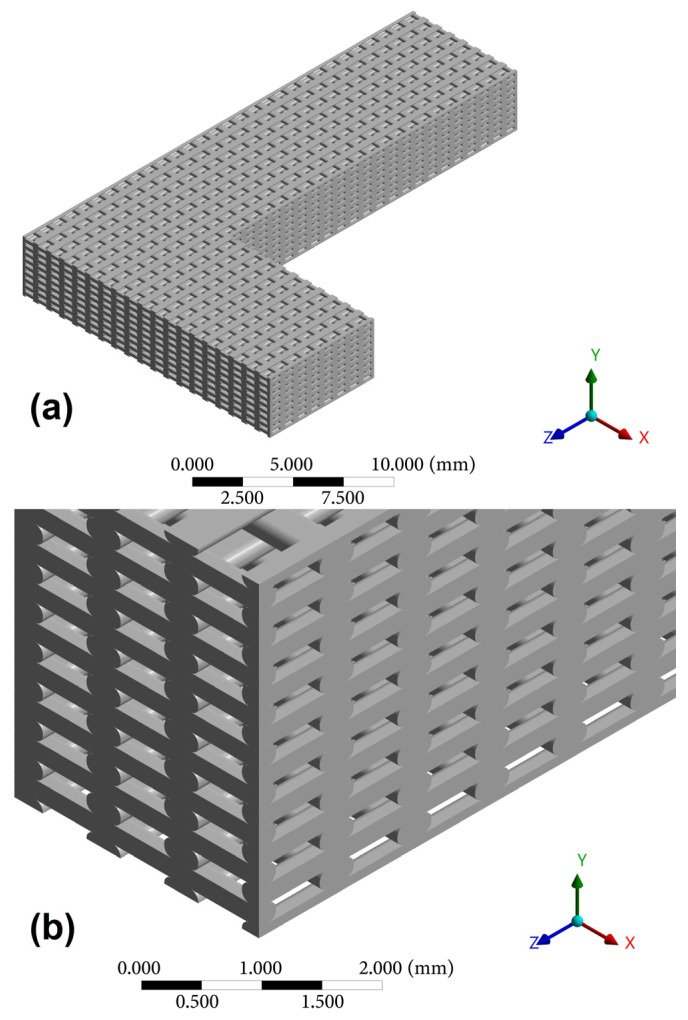
(**a**) Simplified L-beam model; (**b**) Detail of the FDM model framework.

Due to the high computational cost associated with FEM simulation, the support areas were removed, and the edges not considered in the homogenization were trimmed, with the goal of reducing the number of elements required for the simulation. The simplifications made in the model are shown in Figure 12a,b.

As in Section 3.1, a convergence mesh analysis was conducted to determine the maximum element size at which the results are accurate and do not significantly depend on the mesh.

Once the mesh quality was verified, simulations were performed for Tests 1 and 2, applying a displacement of 3 mm to each of the forces defined in Section 2.5. The results obtained from the measurements for points 1, 2, and 3 are presented in Table 4 and Table 5.

The results clearly reflect the influence of porosity on the mechanical behavior, showing a decreasing trend in reaction forces and rotation angles as the void volume increases. This phenomenon has been widely documented by Barzgar et al. (2024) and Zhen et al. (2010) [7,8], who concluded that the loss of structural continuity associated with higher porosity levels significantly compromises the overall stiffness of scaffolds.

#### 3.2.2. Virtual Test of Effective Medium

**Figure 13:**
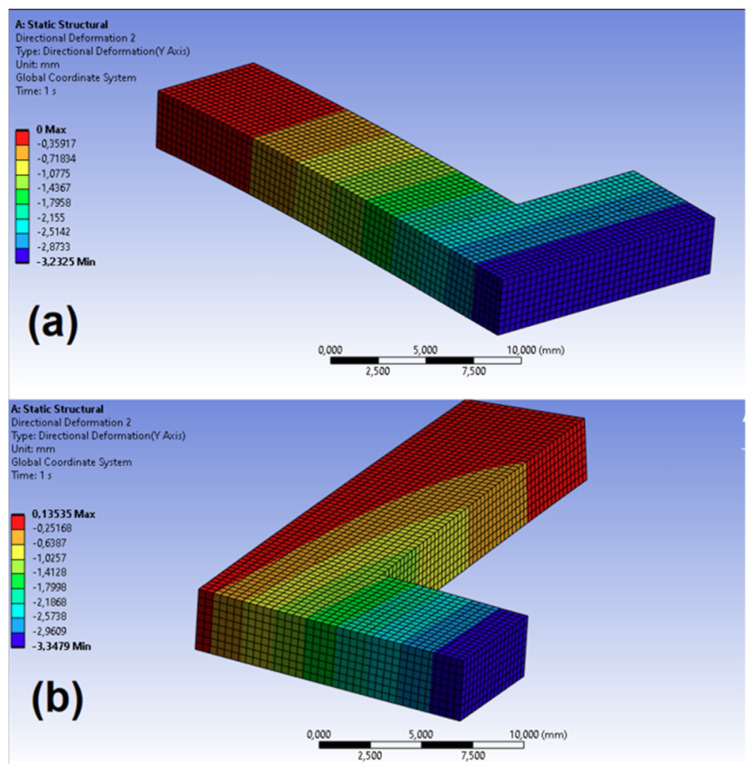
Displacement field in the direction of axis 2 in the virtual test for the effective medium: (**a**) Test 1; (**b**) Test 2.

The virtual test for the effective medium was designed to optimize the determination of elastic constants based on the measurements obtained from the virtual FDM test. To ensure consistency between both models, the same boundary conditions, mesh size (0.07 mm), and external dimensions were used in each test. In this case, the mechanical properties were those obtained from the homogenized model (Table 3). Figure 13 presents the geometry, mesh, and displacement field distribution in the direction of axis *2* for Tests 1 and 2 conducted with the effective medium model and test case 1_50%.

The measurements obtained in both virtual tests (FDM and the effective medium model) were compared to verify the validity of the simplified model. The comparative results are detailed in Table 6 and Table 7 for Test case 1 (1_50%).

The comparison of the results presented in Table 6 and Table 7 revealed a high level of agreement between the virtual test of the effective medium and the FDM simulation, with differences below 1.6% in most measurements, validating the accuracy of the simplified orthotropic model. This consistency supports the findings of Gonabadi et al. (2022) [36], who highlighted the effectiveness of homogenized models in predicting elastic properties of FDM structures under complex loading conditions. The discrepancy observed in the reaction force during Test 2 (−6.15%) may be attributed to variations in boundary conditions, as noted by Luciano and Sacco (1998) [46] in unit cell-based models. Nevertheless, the model accurately reproduced the point displacements (0% error), confirming its ability to capture relevant local deformations.

### 3.3. Optimization

Once the measurements for both tests were verified, an optimization process was implemented to estimate the elastic constants of the parts manufactured using FDM. The measurements obtained from the FDM Model were used as target values, and upper and lower constraints were included to improve accuracy. The MOGA method configured 400 initial samples, 150 samples per iteration, generating three candidates per cycle.

The optimization process for each porosity test case required approximately 3 to 5 h of computational time, using an Intel Core i9 processor (12th Gen) with 32 GB RAM, leveraging multi-core parallelization where possible.

#### 3.3.1. Test 1

The results of Test 1 for the test cases studied are detailed below. An orthotropic base material is initially assigned to each test case, with the initial elastic constants presented in Table 8. The target values and constraints defined for the optimization, including upper and lower limits as specified in Section 2.4, are also provided.

The optimization simulations converge for points 1, 2, and 3 after 1089, 1645, and 1226 iterations, respectively. Consequently, the acceptance criterion for the candidates is applied.

**Figure 14:**
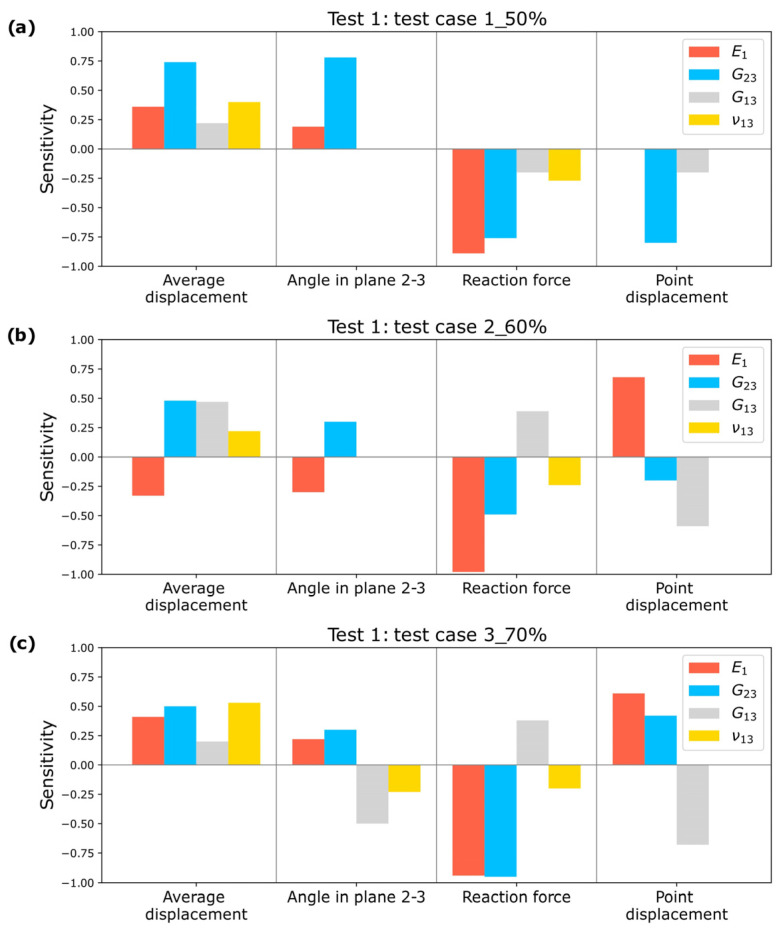
Sensitivity Study for Test 1: (**a**) Test case 1_50%; (**b**) Test case 2_60%; (**c**) Test case 3_70%.

A sensitivity analysis is conducted, as shown in Figure 14, demonstrating that the input parameters significantly impact the estimated elastic constants. This validates the selection of measurements for the optimization process.

**Figure 15:**
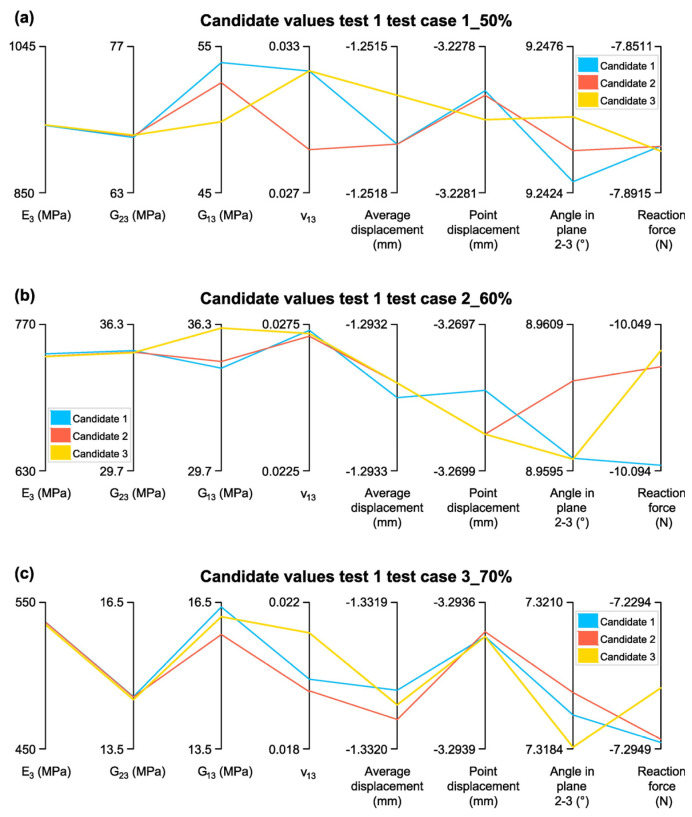
Candidate Values for Test 1: (**a**)Test case 1_50%; (**b**) Test case 2_60%; (**c**) Test case 3_70%.

Once the convergence and sensitivity of the parameters were confirmed, it was verified that the estimated elastic constants were not at the extremes of their evaluation range, thereby discarding non-representative values. Figure 15 presents the three selected candidates for the test cases studied, along with their estimated elastic constants and target values.

The obtained elastic constants are validated by comparing them with the homogenization results (indicated in Table 3, Section 3.1) for the test cases studied. This comparison is presented in Table 9.

The validation of Candidate 1 for the three test cases reveals a strong agreement between the estimated elastic constants and the homogenization results, particularly for $E_{3}$ and $G_{23}$, while $\nu_{13}$ exhibits greater variability. In Test case 1_50%, the estimated $E_{3}$ value of 940.26 MPa is only 1.3% higher than the homogenized value (928.20 MPa), demonstrating high accuracy. Similarly, $G_{23}$ differs by just 0.43%, while $G_{13}$ shows a slightly higher discrepancy of 1.76%, remaining within acceptable limits (lower than 5%). However, ν13 deviates by 3.84%, suggesting some sensitivity to input variations.

In Test case 2_60%, the accuracy of $E_{3}$ remains high, with a difference of 1.2% from the homogenized value (733.14 MPa vs. 741.94 MPa). $G_{23}$ and $G_{13}$ show differences of 1.91% and 9.97%, respectively, indicating good overall agreement but a slightly higher deviation in $G_{13}$, possibly due to anisotropic effects or variations in material distribution. $\nu_{13}$, however, presents a 13.84% difference, reinforcing the trend observed in Test case 1 that Poisson’s ratio is more sensitive to changes in input parameters.

For Test case 3_70%, the estimated $E_{3}$ is 536.61 MPa, differing by only −1.29% from the homogenized value (533.14 MPa), confirming the accuracy of the optimization approach. $G_{23}$ remains consistent, with a minimal deviation of 0.47%, while $G_{13}$ shows a slightly larger but still reasonable difference of 8.59%. However, $\nu_{13}\;$again exhibits significant variation, with a 11.08% discrepancy, further highlighting the challenge of precisely predicting Poisson’s ratio.

Overall, Candidate 1 demonstrates an acceptable alignment with the homogenized values for $E_{3}$ and $G_{23}$ across all test cases, confirming the reliability of the optimization approach in estimating elastic constants. The higher discrepancies observed in $\nu_{13}\;$and, to a lesser extent, $G_{13}$, suggest that these parameters may require further refinement or additional constraints to improve prediction accuracy.

#### 3.3.2. Test 2

In Test 2, the remaining elastic constants $E_{2}$, $\nu_{12}$, and $\nu_{23}$ are estimated. The target value for each measurement point corresponds to the one obtained from the respective FDM Model (Table 8), with its corresponding limits as described in Section 2.4.

**Figure 16:**
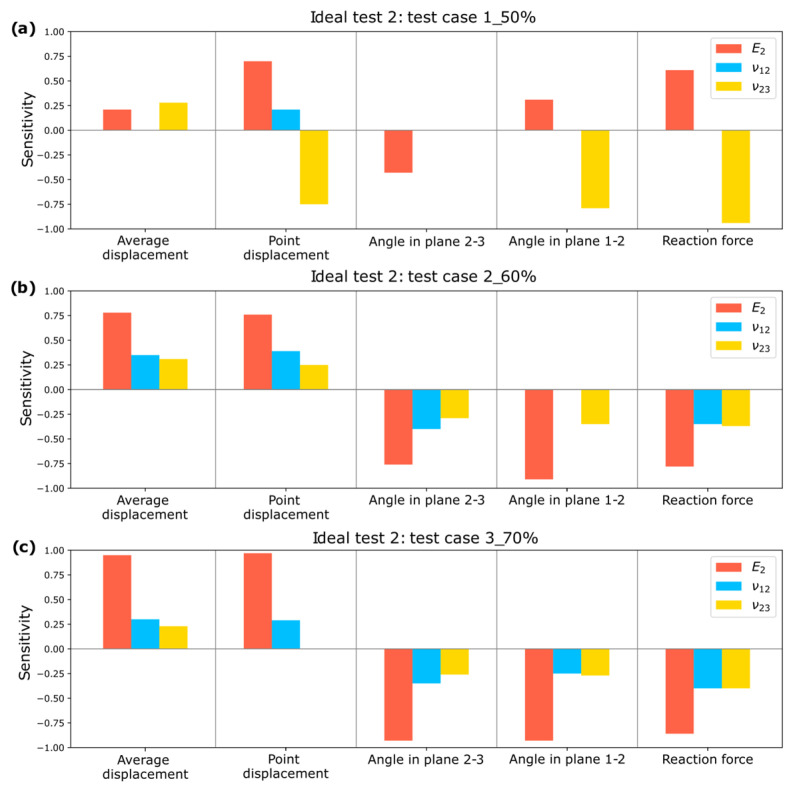
Sensitivity Study for Test 2: (**a**) Test case 1_50%; (**b**) Test case 2_60%; (**c**) Test case 3_70%.

A sensitivity analysis is performed for the optimizations conducted in Test 2, and the results for Test cases 1_50%, 2_60%, and 3_70% can be observed in Figure 16.

It is observed that all input parameters influence the output parameters, confirming that the selected measurements enable the estimation of elastic constants in Test 2.

**Figure 17:**
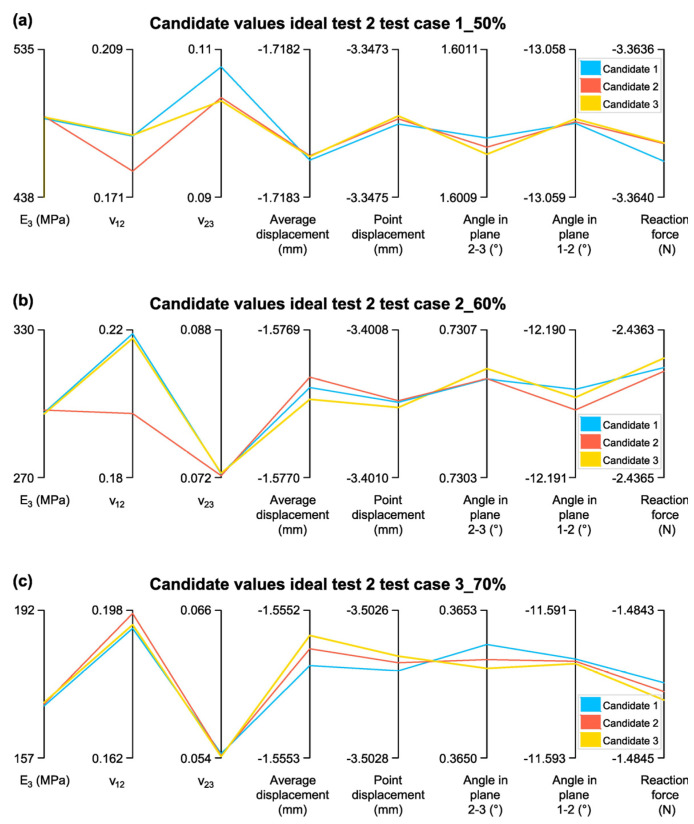
Candidate Values for Test 2: (**a**) Test case 1_50%; (**b**) Test case 2_60%; (**c**) Test case 3_70%.

Once the convergence and sensitivity of the parameters are verified, an analysis is conducted to determine whether the estimated elastic constants fall at the extremes of their evaluation range. If so, they would be discarded. Figure 17 presents the selected candidates along with their respective elastic constants and measurement values.

In Figure 17a, the elastic constants were not estimated at the extremes of their range, confirming the validity of the results. However, in Figure 17b,c, *ν*_23_ was estimated at the extreme of its range. While this is not the ideal outcome, the candidates remain acceptable for further analysis.

The obtained elastic constants are validated by comparing them with the homogenization results from points 1, 2, and 3, as shown in Table 10.

The estimated elastic constants were validated by contrasting them with the homogenization results (Table 10). The discrepancies between the estimated and homogenized values remained within acceptable margins for $E_{2}$ and $\nu_{12}$, whereas $\nu_{23}$ exhibited greater deviations, confirming the trend observed in the sensitivity analysis.

For Test case 1_50%, $E_{2}$ was estimated at 489.55 MPa, showing a minimal deviation of 0.78% from the homogenized value (485.76 MPa), indicating a strong agreement. $\nu_{12}$ was slightly lower than expected, with a −2.88% difference, which remains within acceptable limits. However, $\nu_{23}$ exhibited a higher deviation of 7.26%, suggesting a greater sensitivity of Poisson’s ratios to input variations.

In Test case 2_60%, the estimated $E_{2}$ value of 296.32 MPa showed a −4.37% deviation, slightly higher than in Test case 1 but still maintaining good accuracy; $\nu_{12}$, however, presented a larger variation of 15.86%, highlighting its greater sensitivity to input conditions. The $\nu_{23}$ discrepancy was also notable at −9.71%, reinforcing the difficulty in accurately estimating this parameter.

For Test case 3_70%, $E_{2}$ was estimated at 169.35 MPa, showing a −3.23% deviation, which confirms the high accuracy of the estimation. $\nu_{12}$ varied by 3.41%, which is a better agreement compared to Test case 2, while $\nu_{23}$ presented the largest deviation (−10.03%), further emphasizing the challenges in accurately predicting Poisson’s ratio.

**Figure 18:**
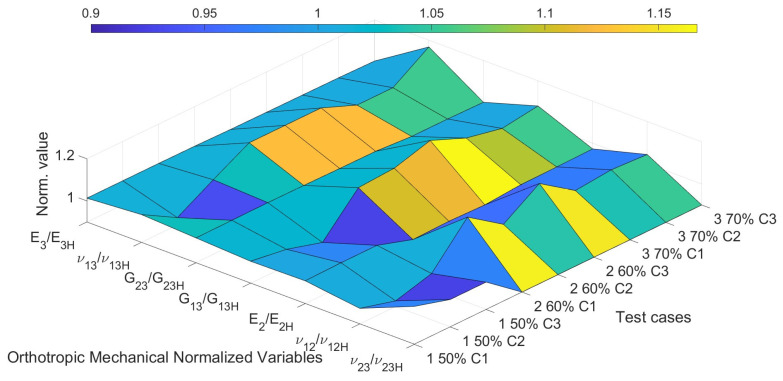
3D surface plot of normalized orthotropic elastic constants (X/X_h_) estimated by MOGA.

Figure 18 presents a three-dimensional surface plot that visualizes the normalized values of the orthotropic elastic constants estimated by the optimization algorithm (MOGA), in relation to their respective reference values obtained through homogenization. Each point on the surface corresponds to a normalized ratio of the form ***X****/**X**_H_*, where *X* denotes one of the seven mechanical parameters of interest, $E_{3},\;\nu_{13},\;G_{23},\;G_{13},\;E_{2},\;\nu_{12},\;\nu_{23}$, and *X_H_* represents its homogenized reference value.

The *x*-axis groups these seven normalized mechanical variables, while the *y*-axis organizes the different test cases and their respective candidate solutions (e.g., “1 50% C1” corresponds to Candidate 1 for the test case with 50% porosity). The *z*-axis shows the magnitude of the normalized value, where a value close to 1.0 indicates strong agreement between the estimated and reference properties. It can be observed that most elastic constants lie within a ±10% range from their reference values, which reinforces the consistency and reliability of the proposed optimization framework.

Finally, Table 11 presents the percentage differences related to Candidate 1 for the material’s mechanical properties obtained through virtual testing and topology optimization compared to a homogenization method.

Although most parameters exhibit low (lower than 5%) and acceptable (lower than 10%) percentage errors, the Poisson’s ratios (especially ν_12_ and ν_23_) show greater sensitivity and deviation, which may require additional adjustments in the geometric design or optimization strategy to improve their accuracy.

## 4. Conclusions

In this study, a method for the characterization of materials fabricated via FDM was developed, minimizing the need for extensive experiments, highlighting its potential applications in biomaterials development. Moreover, by integrating virtual testing, homogenization, and optimization under biological and geometric constraints, the methodology has proven effective and may serve as a reference framework for future scaffold design in tissue engineering.

Based on these findings, several conclusions have been identified, emphasizing the validity and limitations of the proposed approach, as detailed below:

- The results of the virtual test for the FDM model were compared with the effective medium model, revealing differences below 1.6% in most measurements. However, a larger discrepancy was observed in the reaction force for Test 2 (−6.15%), possibly due to variations in boundary conditions or load distribution in the simplified model. This confirms that the virtual test is a valid tool for estimating mechanical properties, although certain measurements exhibit greater sensitivity to model test case.
- The elastic constants estimated through the optimization process were validated by comparison with the results obtained from homogenization. It was observed that $E_{2}$ and $\nu_{12}$ exhibited acceptable differences, whereas $\nu_{23}$ showed greater discrepancies. This confirms the trend identified in the sensitivity analysis, indicating that certain elastic constants are more challenging to estimate with precision.
- The elastic constant values changed with the analyzed porosity levels (50%, 60%, and 70%), confirming the influence of printing parameters on the material’s mechanical response. It was observed that a higher porosity level led to a reduction in $E_{2}$ and $G_{23}$, which is consistent with previous studies on the relationship between cellular structure and mechanical properties.
- The multi-objective genetic algorithm (MOGA) effectively optimized the elastic constants, with convergence times varying based on the test case and test applied. Sensitivity analysis confirmed that all input parameters had a direct impact on the output, validating the suitability of the selected measurements for estimating elastic constants. Moreover, the estimated values remained within the expected range, except for specific cases such as $\nu_{23}$ in test cases 2_60% and 3_70%.

The virtual testing and optimization-based methodology has proven to be effective for characterizing 3D-printed materials with cellular structures. However, future research should focus on refining the estimation of Poisson’s ratios and exploring alternative optimization methods to minimize discrepancies in highly sensitive parameters such as $\nu_{23}$.

Although the proposed virtual optimization framework was validated based on mechanical properties and design constraints relevant to tissue engineering, it is acknowledged that such virtual assessment cannot fully replace direct experimental validation. Therefore, future studies will include mechanical testing of physical prototypes, as well as in vitro evaluations, to confirm the structural integrity and functional viability of the proposed designs, especially considering potential artifacts associated with the 3D printing process, such as surface irregularities and edge effects, which may influence the mechanical response but are not fully captured by theoretical modeling.

This work lays the foundation for a mechanical characterization approach based on virtual testing and multi-objective optimization in 3D-printed orthotropic structures. Nevertheless, several opportunities for improvement have been identified that could strengthen and expand the proposed methodology. First, the incorporation of regularization terms (such as Tikhonov regularization) could help stabilize the identification of sensitive parameters like ν_23_ and constrain the solutions to physically plausible ranges. Likewise, the application of global sensitivity analysis methods, such as Sobol indices, is recommended to prioritize experimental constraints with higher informational contribution (e.g., in-plane rotations) and eliminate those with low sensitivity, thereby improving model efficiency.

Additionally, it would be valuable to compare the performance of the MOGA with other evolutionary algorithms, such as NSGA-II, to validate its robustness in addressing complex nonlinear problems.

On the experimental side, we propose the development of mechanical tests on FDM-printed L-shaped beams, incorporating digital image correlation (DIC) techniques to validate the simulated displacement fields. Complementary targeted experiments, such as biaxial tensile tests, could be designed for parameters that are difficult to estimate (e.g., ν_23_), in order to obtain more accurate empirical references.

Finally, future research could incorporate additional objectives into the optimization process, such as pore connectivity quantified via micro-computed tomography (CT) or fluid shear stress obtained through computational fluid dynamics (CFD), thus enabling the integration of biological constraints directly into the design process.

## Figures and Tables

**Table 1:** Test Cases for scaffold mechanical properties.

Test Cases	Layer Height/Width (mm)	Porosity (%)
1_50%	0.2/0.4	50
2_60%	0.2/0.4	60
3_70%	0.2/0.4	70

**Table 2:** Displacement boundary conditions for a unit cell with dimensions a, b, and c.

Deformation	Displacement	A_1_	A_2_	B_1_	B_2_	C_1_	C_2_
$\varepsilon_{11}=0.01$	u	b × 0.01	0	Free	Free	Free	Free
	v	Free	Free	Free	Free	0	0
	w	0	Free	Free	Free	0	0
$\varepsilon_{22}$ =0.01	u	Free	a × 0.01	Free	Free	Free	Free
	v	Free	Free	Free	Free	0	0
	w	0	Free	Free	Free	0	0
$\varepsilon_{33}=0.01$	u	Free	0	Free	Free	Free	Free
	v	Free	Free	Free	Free	0	0
	w	0	Free	Free	Free	0	0
$\gamma_{12}=0.01$	u	b × 0.005	0	a × 0.005	0	Free	Free
	v	Free	Free	Free	Free	Free	Free
	w	Free	Free	Free	Free	Free	Free
$\gamma_{13}=0.01$	u	Free	c × 0.005	Free	0	c × 0.01	Free
	v	Free	Free	Free	Free	Free	Free
	w	Free	Free	Free	Free	Free	Free
$\gamma_{23}=0.01$	u	Free	Free	Free	c × 0.005	Free	b × 0.005
	v	Free	Free	Free	Free	Free	Free
	w	Free	Free	Free	Free	Free	Free

**Table 3:** Orthotropic mechanical properties obtained from the homogenization process.

Test Cases	$E_{1}$ (MPa)	$E_{2}$ (MPa)	$E_{3}$ (MPa)	$\nu_{12}$	$\nu_{13}$	$\nu_{23}$	$G_{21}$ (MPa)	$G_{13}$ (MPa)	$G_{23}$ (MPa)
**1_50%**	928.20	485.76	928.32	0.19	0.031	0.10	67.17	53.73	67.17
**2_60%**	733.14	309.85	733.09	0.19	0.024	0.08	34.53	31.06	34.47
**3_70%**	543.65	175.00	543.68	0.18	0.018	0.06	13.92	14.05	13.92

**Table 4:** Measurements for Test 1 for Test cases 1_50%, 2_60%, and 3_70%.

Measurement	1_50%	2_60%	3_70%
**Average displacement (mm)**	−1.23	−1.29	−1.34
**Point displacement (mm)**	−3.23	−3.28	−3.30
**Angle in plane 2**–**3 (°)**	9.25	8.96	7.3
**Reaction force (N)**	−7.88	−10.1	−7.48

**Table 5:** Measurements for Test 2 for Test cases 1_50%, 2_60%, and 3_70%.

Measurement	1_50%	2_60%	3_70%
**Average displacement (mm)**	−1.72	−1.59	−1.56
**Point displacement (mm)**	−3.34	−3.39	−3.49
**Angle in plane 2**–**3 (°)**	1.62	0.76	0.38
**Angle in plane 1**–**2 (°)**	−12.95	−12.01	−11.52
**Reaction force (N)**	−3.58	−2.62	−1.60

**Table 6:** Comparison of Virtual FDM Test and Virtual Effective Model Test During Test 1 (1_50%).

Measurement	FDM	Effective Medium	Percentage Difference (%)
**Average displacement (mm)**	−1.23	−1.25	1.63
**Point displacement (mm)**	−3.23	−3.23	0.00
**Angle in plane 2**–**3 (°)**	9.25	9.24	−0.11
**Reaction force (N)**	−7.88	−7.77	−1.40

**Table 7:** Comparison of Virtual FDM Test and Virtual Effective Model Test During Test 2 (1_50%).

Measurement	FDM	Effective Medium	Percentage Difference (%)
**Average displacement (mm)**	−1.72	−1.72	0.00
**Point displacement (mm)**	−3.34	−3.35	0.30
**Angle in plane 2**–**3 (°)**	1.62	1.60	−1.23
**Angle in plane 1**–**2 (°)**	−12.95	−13.06	0.85
**Reaction force (N)**	−3.58	−3.36	−6.15

**Table 8:** Initial Values of Mechanical Properties for the base Material.

Test Case	Density (kg/m^3^)	$E_{1}$ (MPa)	$E_{2}$ (MPa)	$E_{3}$ (MPa)	$\nu_{12}$	$\nu_{13}$	$\nu_{23}$	$G_{21}$ (MPa)	$G_{13}$ (MPa)	$G_{23}$ (MPa)
**1_50%**	620	950	500	950	0.2	0.1	0.03	70	50	70
**2_60%**	496	700	300	700	0.2	0.1	0.02	33	33	33
**3_70%**	372	500	200	500	0.2	0.1	0.05	15	15	15

**Table 9:** Validation of Elastic Constants in Test 1.

Test Case	Candidate	$E_{3}$ (MPa)	$\nu_{13}$	$G_{23}$ (MPa)	$G_{13}$ (MPa)
**1_50%**	Candidate 1	940.26	0.032	68.35	53.96
	Candidate 2	940.22	0.029	68.45	52.53
	Candidate 3	940.34	0.032	68.53	49.87
	**Homogenization**	**928.20**	**0.031**	**67.17**	**53.73**
	Difference (Candidate 1)	1.30%	3.84%	0.43%	1.76%
	Difference (Candidate 2)	1.30%	−7.17%	−2.23%	1.91%
	Difference (Candidate 3)	1.31%	4.79%	−7.19%	2.02%
**2_60%**	Candidate 1	741.94	0.027	35.12	34.33
	Candidate 2	739.64	0.027	35.05	34.63
	Candidate 3	739.27	0.027	35.03	36.14
	**Homogenization**	**733.14**	**0.024**	**34.47**	**31.06**
	Difference (Candidate 1)	1.20%	13.84%	1.91%	9.97%
	Difference (Candidate 2)	0.89%	13.27%	1.69%	11.52%
	Difference (Candidate 3)	0.84%	13.33%	1.64%	16.35%
**3_70%**	Candidate 1	536.61	0.019	14.56	16.40
	Candidate 2	536.54	0.019	14.56	15.84
	Candidate 3	534.87	0.021	14.51	16.20
	**Homogenization**	**533.14**	**0.018**	**14.50**	**15.00**
	Difference (Candidate 1)	−1.29%	11.08%	0.47%	8.59%
	Difference (Candidate 2)	−1.31%	8.68%	0.43%	4.85%
	Difference (Candidate 3)	−1.61%	17.67%	0.07%	7.26%

**Table 10:** Validation of Elastic Constants in Test 2.

Test Case	Candidate	$E_{2}$ (MPa)	$\nu_{12}$	$\nu_{23}$
**1_50%**	Candidate 1	489.55	0.187	0.108
	Candidate 2	491.11	0.178	0.103
	Candidate 3	491.10	0.187	0.103
	**Homogenization**	**485.76**	**0.192**	**0.100**
	Difference (Candidate 1)	0.78%	−2.88%	7.26%
	Difference (Candidate 2)	1.10%	−7.57%	3.10%
	Difference (Candidate 3)	1.10%	−2.76%	2.66%
**2_60%**	Candidate 1	296.32	0.219	0.072
	Candidate 2	297.42	0.197	0.072
	Candidate 3	295.89	0.218	0.072
	**Homogenization**	**309.85**	**0.189**	**0.080**
	Difference (Candidate 1)	−4.37%	15.86%	−9.71%
	Difference (Candidate 2)	−4.01%	4.42%	−9.95%
	Difference (Candidate 3)	−4.50%	15.20%	−9.62%
**3_70%**	Candidate 1	169.35	0.194	0.054
	Candidate 2	169.79	0.197	0.054
	Candidate 3	170.19	0.195	0.054
	**Homogenization**	**175.00**	**0.187**	**0.060**
	Difference (Candidate 1)	−3.23%	3.41%	−10.03%
	Difference (Candidate 2)	−2.98%	5.32%	−10.28%
	Difference (Candidate 3)	−2.75%	3.85%	−10.42%

**Table 11:** Comparative Analysis of the Error Between the Elastic Constants of the Homogenized Model and the Virtual Testing Method Based on Optimization Criteria.

Parameter	Test Case 1_50%	Test Case 2_60%	Test Case 3_70%
$E_{1}=E_{3}$	1.30%	1.20%	−1.29%
$E_{2}$	0.78%	−4.37%	−3.23%
$\nu_{12}$	−2.88%	15.86%	3.41%
$\nu_{13}$	3.84%	13.84%	11.08%
$\nu_{23}$	7.26%	−9.71%	−10.03%
$G_{12}=G_{23}$	0.43%	1.91%	0.47%
$G_{13}$	1.76%	9.97%	8.59%
